# Fertility‐Sparing Management of Grade 2 Endometrioid Endometrial Adenocarcinoma Without Progesterone Receptor Expression: A Case Report

**DOI:** 10.1155/crog/5582003

**Published:** 2026-03-15

**Authors:** Luiz Felipe Lessa Ortiz, Rafael Dyer Rodrigues de Moraes, Renan Ribeiro e Ribeiro, Caio Bosquê Hidalgo Ribeiro, Amanda Lino De Faria Lessa, Bárbara Bomfim Muniz Moraes, Henrique Cunha Vieira, Giorgio Bogani

**Affiliations:** ^1^ Department of Reproductive Medicine and Oncofertility, GAVVA Clinic, Sao Paulo, Sao Paulo, Brazil; ^2^ CICAP – Oswaldo Cruz German Hospital, Sao Paulo, Brazil; ^3^ IRCCS National Cancer Institute of Milan, Milan, Italy

**Keywords:** conservative treatment, endometrial cancer, endometrial carcinoma, fertility preservation, fertility sparing, hormone receptors, progestin

## Abstract

Managing G2 endometrioid endometrial adenocarcinoma without progesterone receptor (PR) expression presents significant challenges, particularly when considering fertility preservation. Grade 2 tumors fall into an area of uncertainty within fertility‐sparing strategies, making treatment decisions complex and requiring careful individualization. This case report describes a 41‐year‐old patient who, despite PR and PE negativity, responded to conservative management with hysteroscopic resection, a levonorgestrel‐releasing intrauterine device, metformin, and later megestrol, achieving complete histological remission in 15 months. The difficulty in managing G2 tumors lies in their variable behavior, necessitating a multidisciplinary approach, strict monitoring, and adaptability based on treatment response. This case underscores that, although fertility preservation in PR‐negative G2 tumors remains challenging, it is feasible in highly selected cases. The evolving literature suggests that alternative strategies, such as immune modulation and aromatase inhibitors, may further expand options. Individualized management remains crucial, balancing oncological safety with reproductive goals through continuous reassessment and engagement with emerging therapeutic evidence.

## 1. Introduction

Endometrial cancer represents the most common gynecologic malignancy in high‐income countries, with a steadily increasing global incidence largely attributed to the rising prevalence of obesity, metabolic dysfunction, and delayed childbearing. Although it remains the leading gynecologic cancer in developed regions, it is the second most common gynecologic malignancy in many low‐ and middle‐income countries, surpassed only by cervical cancer. In 2022, ~420,368 new cases of uterine corpus cancer were diagnosed worldwide, and notably, up to 14% occurred in women of reproductive age, defined by the World Health Organization as ≤49 years. This epidemiologic shift has profound clinical implications, as an increasing number of young women are diagnosed before completing their reproductive plans [1–3].

The endometrioid subtype accounts for most endometrial cancers diagnosed in younger patients and is typically associated with early‐stage disease and favorable oncologic outcomes. These tumors are frequently estrogen‐driven and often arise in the context of obesity, insulin resistance, polycystic ovary syndrome, and other components of metabolic syndrome [1–4]. Consequently, fertility preservation has become an increasingly relevant goal in the management of selected patients with early‐stage endometrioid endometrial carcinoma. Conservative, fertility‐sparing treatment strategies (primarily based on progestin therapy) have been incorporated into international guidelines for highly selected patients with grade 1, stage IA disease confined to the endometrium [5–13].

However, the extension of fertility‐sparing management beyond low‐grade tumors remains controversial. In particular, endometrioid grade 2 endometrial carcinoma represents a gray zone in clinical practice. Although grade 2 tumors exhibit potentially worse prognostic biological behavior compared to grade 1 tumors, emerging evidence suggests that a subset of carefully selected patients with grade 2 disease may achieve acceptable oncologic outcomes under conservative management, especially when modern molecular classification is incorporated into risk stratification. Nevertheless, data remain limited, and the safety of this approach continues to be debated [5, 8, 14–16].

A critical determinant of response to conservative treatment is hormonal receptor status, particularly progesterone receptor (PR) expression. Progestin‐based therapy exerts its antitumor effect through PR‐mediated pathways, promoting cellular differentiation, inhibiting proliferation, and counteracting estrogen‐driven growth. Multiple studies have consistently demonstrated that PR positivity is strongly associated with higher rates of complete response, longer disease‐free intervals, and lower recurrence rates following fertility‐sparing treatment. Conversely, the absence of PR expression has been associated with reduced responsiveness to progestin therapy, earlier treatment failure, and increased risk of disease persistence or progression, raising significant concerns regarding oncologic safety in this subgroup [5, 8, 14–16].

The management of young patients with endometrioid grade 2 endometrial cancer lacking PR expression is therefore particularly challenging. These patients fall outside conventional eligibility criteria for fertility preservation, yet often present with a strong desire to maintain reproductive potential. The absence of robust prospective data, combined with the evolving role of molecular classification and assisted reproductive technologies, necessitates a highly individualized, multidisciplinary approach. Decisions must balance oncologic safety with reproductive goals, integrating histopathologic features, receptor status, molecular profile, patient preferences, and the feasibility of close surveillance and timely definitive surgery [5, 8, 14–16].

In this context, the present case report illustrates the complexities and clinical dilemmas inherent in fertility‐sparing management of endometrioid grade 2 endometrial carcinoma without PR and estrogen receptor (ER) expression. It underscores the limitations of current evidence, highlights the importance of personalized decision‐making, and contributes to the ongoing discussion regarding the boundaries of conservative treatment in the era of molecularly informed gynecologic oncology [5, 8, 14–16].

## 2. Case Report

A 41‐year‐old nulligravid woman was diagnosed with an extensive grade 2 (G2) endometrioid endometrial adenocarcinoma lacking PR expression and ER, as confirmed by immunohistochemistry. Molecular testing revealed no specific alterations, and the tumor was classified as no specific molecular profile (NSMP)—Supporting Information 1: Figure S2. The patient expressed a strong desire to preserve fertility and was referred for evaluation of fertility‐sparing treatment. Pelvic magnetic resonance imaging (MRI) demonstrated an endometrial lesion confined to the uterine cavity, without evidence of myometrial invasion, despite extensive involvement of the endometrial surface.

After multidisciplinary discussion, conservative treatment was initiated with hysteroscopic resection of the endometrial lesion followed by insertion of a levonorgestrel‐releasing intrauterine device, combined with metformin therapy. This approach was based on selected reports in the literature suggesting potential benefit in carefully selected cases, as well as the patient’s strong desire for fertility preservation. The patient was closely monitored with serial pelvic MRI and hysteroscopic endometrial biopsies every 3–4 months. In addition, computed tomography (CT) of the chest was performed during follow‐up and demonstrated no evidence of extra‐uterine disease.

## 3. Outcome and Evolution

After 6 months of treatment, significant lesion reduction was observed, though residual disease remained. The option of continuing conservative treatment was discussed based on continuous literature review and promising new therapeutic modalities. The patient chose to extend the conservative treatment with strict follow‐up.

After 12 months, the patient achieved complete macroscopic hysteroscopic remission of the lesion. Targeted endometrial biopsies of the anterior, posterior, right lateral, and left lateral uterine walls were performed, revealing a microscopic residual focus in only one of the sampled areas. Following a new multidisciplinary discussion and approval by the institutional ethics committee, megestrol acetate at a dose of 160 mg was introduced, in combination with maintenance of the levonorgestrel‐releasing intrauterine system and serial endometrial biopsies.

At 15 months from the initiation of treatment, the patient achieved complete disease remission (Supporting Information 1: Figure S1), including histological remission. She has remained in complete remission, including histopathological remission, to date (36 months after initial treatment).

The patient subsequently initiated an assisted reproduction program with oocyte donation due to low ovarian reserve, after three unsuccessful attempts to obtain autologous embryos, and is currently scheduled for embryo transfer.

## 4. Discussion

Conservative management of endometrioid endometrial adenocarcinoma is traditionally reserved for low‐grade tumors expressing hormone receptors, particularly PRs, as PR positivity is strongly associated with higher response rates to progestin‐based therapy [5, 17]. The absence of PR expression and PE expression, as observed in this case, represents a significant clinical challenge and has been consistently associated with lower complete response rates, increased disease persistence, and a higher risk of recurrence following fertility‐sparing treatment [8, 17].

Nevertheless, emerging evidence suggests that fertility‐sparing treatment may still be considered in carefully selected patients even in the absence of PR expression, provided that strict eligibility criteria, multidisciplinary management, and close surveillance are ensured [11, 17]. This approach becomes particularly controversial in patients with grade 2 endometrioid tumors, in whom oncologic safety is less well established and available data support feasibility only in highly selected cases [11, 16]. In this context, PR negativity further increases uncertainty, as progestin activity is largely PR‐dependent, rendering grade 2 PR‐negative tumors a biologically ambiguous subgroup requiring heightened caution and predefined exit strategies [14, 16].

Current international guidelines emphasize that fertility preservation should be regarded as a risk‐adapted strategy, primarily limited to patients with stage IA disease confined to the endometrium, without myometrial or lymphovascular invasion, and managed under rigorous follow‐up [5]. In recent years, integration of molecular classification has emerged as a key tool to refine patient selection, particularly in grade 2 disease [11, 14, 15]. Molecular subgroups such as POLE‐ultramutated, MMR‐deficient/MSI‐H, NSMP, and p53‐abnormal tumors provide clinically meaningful risk stratification beyond histologic grade alone. Evidence suggests that fertility‐sparing management may be cautiously considered in selected molecularly favorable grade 2 tumors, whereas p53‐abnormal disease should be excluded from conservative approaches [14, 15].

From a practical standpoint, conservative treatment in biologically uncertain scenarios should be implemented as a structured and time‐limited protocol, rather than an open‐ended trial [5, 11]. This includes accurate imaging‐based staging, expert pathological review, hereditary cancer risk assessment, and clear criteria for treatment discontinuation [5, 11]. Combined strategies—such as hysteroscopic tumor resection followed by systemic progestins and/or a levonorgestrel‐releasing intrauterine system—have been associated with improved local control and acceptable oncologic outcomes in selected cohorts, although responses remain heterogeneous in PR‐negative tumors [8, 12].

Once complete response is achieved, reproductive planning should be prioritized. Several studies indicate higher cumulative pregnancy rates with early use of assisted reproductive technologies, minimizing prolonged exposure to recurrence risk [8, 11, 12]. In patients with grade 2 PR‐negative disease, where the intrinsic risk of persistence or recurrence is higher, this strategy may be particularly relevant [11, 16]. Definitive surgery should be recommended after completion of childbearing or in cases of inadequate response to conservative therapy [5, 11].

In summary, this case illustrates that fertility‐sparing management may be considered, in exceptional circumstances, for patients with PR‐negative, grade 2 endometrioid endometrial carcinoma. Such an approach should be restricted to specialized centers and supported by shared decision‐making, refined molecular and clinicopathologic risk stratification, and strict surveillance protocols [11, 14].

## 5. Conclusion

Conservative management of grade 2 endometrioid endometrial adenocarcinoma without PR expression presents a significant clinical challenge but is feasible in carefully selected cases. This case report emphasizes the critical importance of a multidisciplinary treatment strategy, rigorous follow‐up, and continuous engagement with evolving evidence in the literature to achieve fertility preservation without compromising oncological safety.

## Funding

No funding was received for this manuscript.

## Disclosure

This case report was first presented, with great honor, at the 26th ESGO Congress in Rome, 2025, as an E‐Poster and published in the official proceedings of the congress. It addresses a highly relevant and current topic: fertility preservation in a patient with endometrial cancer. The abstract can be accessed via the following link: https://www.international-journal-of-gynecological-cancer.com/article/S1048-891X(24)21376-1/abstract [18].

## Conflicts of Interest

The authors declare no conflicts of interest.

## Supporting Information

Additional supporting information can be found online in the Supporting Information section.

## Supporting information


**Supporting Information 1** Figure S2. The figure illustrates histological sections of grade 2 endometrioid endometrial adenocarcinoma. Hematoxylin and eosin (H&E) staining demonstrates a transition from glandular areas to nonsquamous solid areas; in another field, a solid area with central necrosis is evident. Immunohistochemistry reveals a molecular profile with preserved nuclear expression of DNA mismatch repair proteins (MMR‐proficient/stable) and negative expression of estrogen and progesterone receptors.

**Supporting Information 2** Figure S1. The image presents two sagittal T2‐weighted MRI sequences of the abdomen and pelvis, comparing the pretreatment state (on the left) and the posttreatment state (on the right). In the pretreatment image, a voluminous lesion is observed in the endometrial cavity, with a high T2 signal, suggesting endometrial neoplasia. In the posttreatment scan, there is a significant reduction or disappearance of the lesion, indicating a positive therapeutic response. Additionally, there appears to be a reduction in endometrial thickening and an improvement in the uterine contour. These images are essential for monitoring treatment efficacy and guiding future strategies for fertility preservation and oncologic control.

**Supporting Information 3** Video 1. Fertility‐sparing treatment for a patient with endometrial cancer G2—hormone receptor negative and extensive intracavitary lesion—with complete response—the video presents the case of a patient diagnosed with grade 2 endometrioid adenocarcinoma of the endometrium, negative for hormone receptors. Initially, a hysteroscopic resection of the lesion—confined to but extensive within the endometrium—was performed, followed by insertion of a Mirena IUD for levonorgestrel release. After 18 months of conservative treatment, repeat hysteroscopy with endometrial biopsy showed no evidence of disease for the second time, confirming the efficacy of the fertility‐sparing approach.

## Data Availability

The data that support the findings of this study are available from the corresponding author upon reasonable request.
