# Supplementary figures and images for: Fertility‐Sparing Management of Grade 2 Endometrioid Endometrial Adenocarcinoma Without Progesterone Receptor Expression: A Case Report
Source: Case Rep Obstet Gynecol. 2026 Mar 15;2026:5582003. doi: 10.1155/crog/5582003 (PMC13107955; doi:10.1155/crog/5582003)

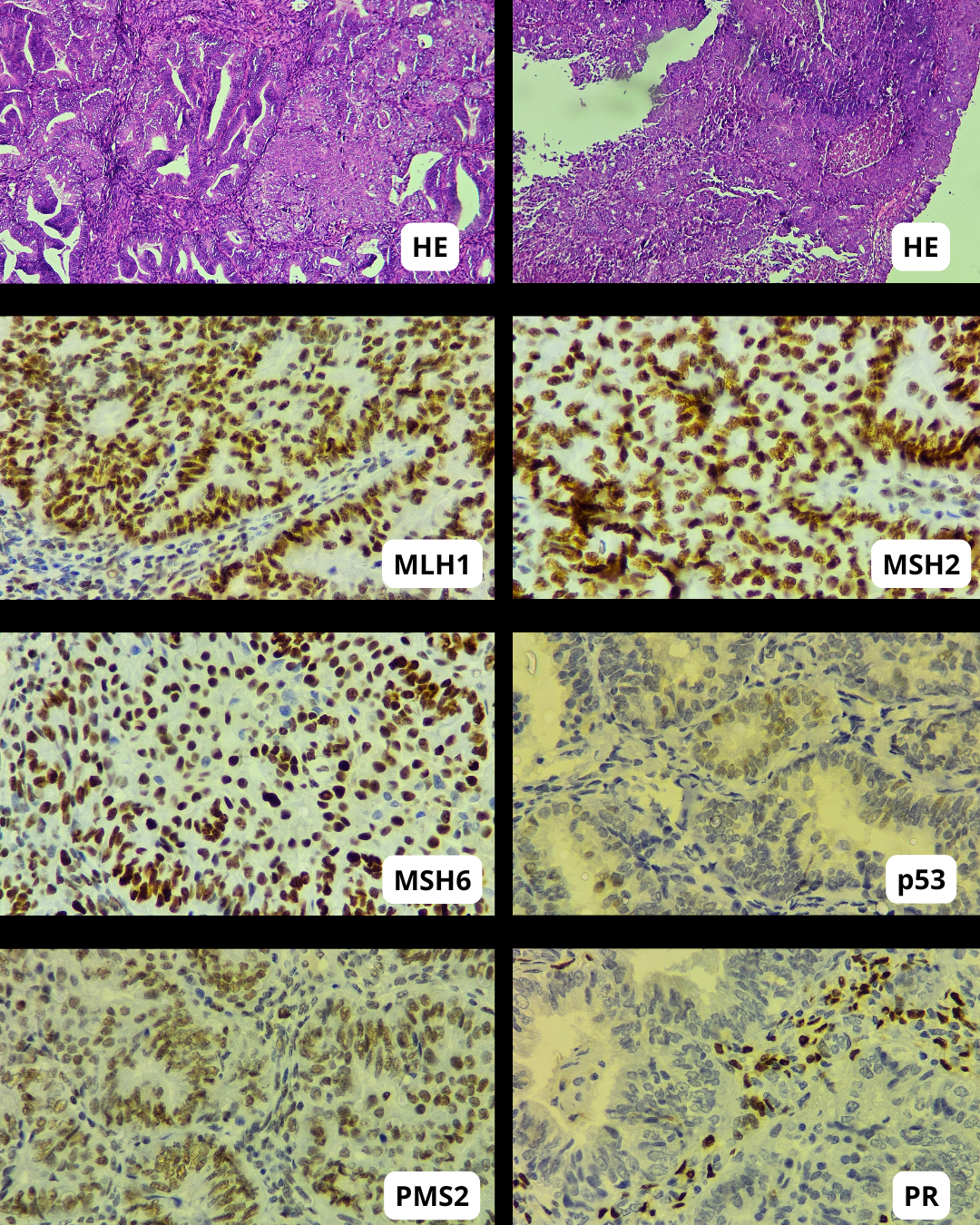

Supplement: Supplementary file 1 — Supporting Information 1 Figure S2. The figure illustrates histological sections of grade 2 endometrioid endometrial adenocarcinoma. Hematoxylin and eosin (H&E) staining demonstrates a transition from glandular areas to nonsquamous solid areas; in another field, a solid area with central necrosis is evident. Immunohistochemistry reveals a molecular profile with preserved nuclear expression of DNA mismatch repair proteins (MMR‐proficient/stable) and negative expression of estrogen and progesterone receptors. [file CROG-2026-5582003-s001.png]

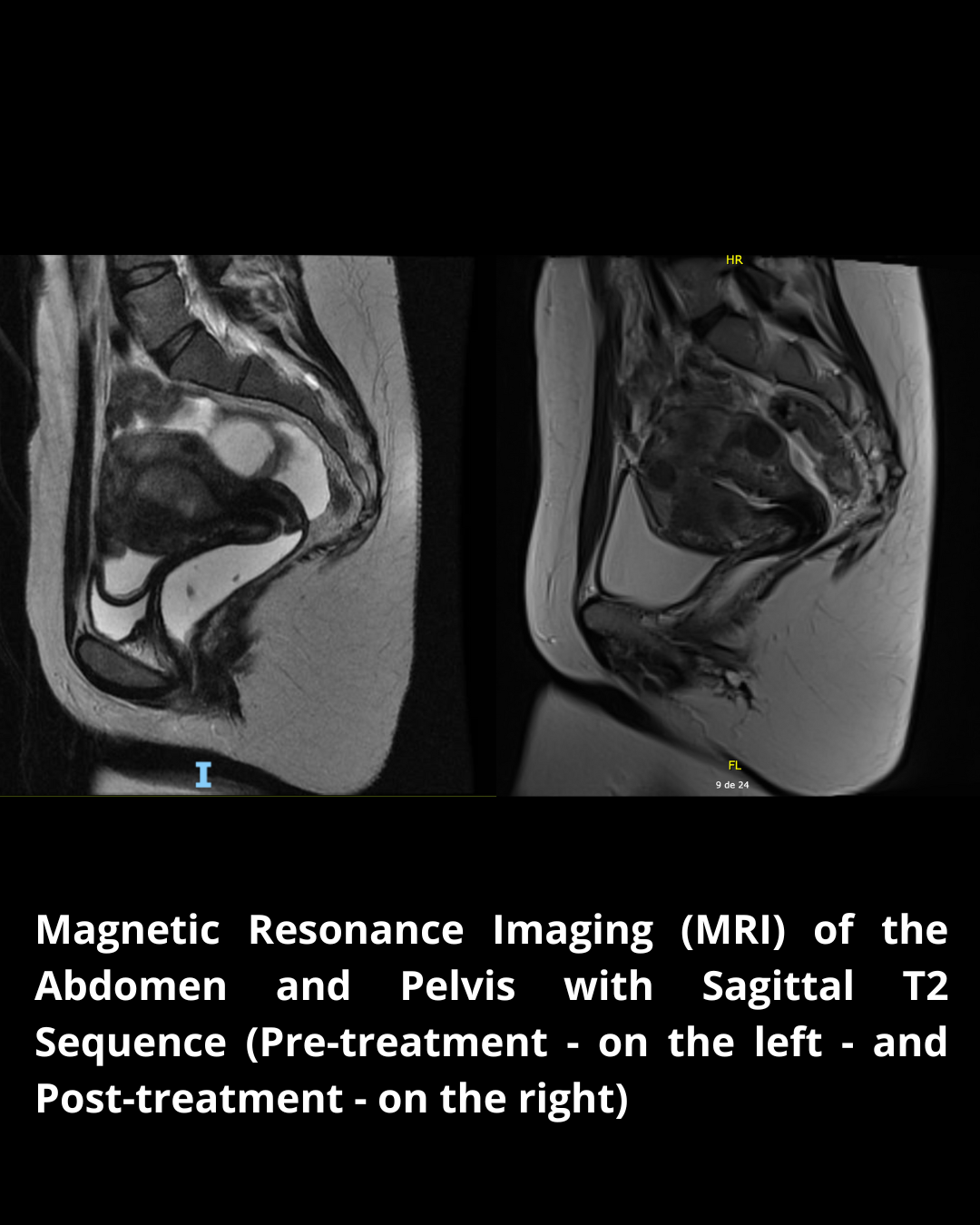

Supplement: Supplementary file 2 — Supporting Information 2 Figure S1. The image presents two sagittal T2‐weighted MRI sequences of the abdomen and pelvis, comparing the pretreatment state (on the left) and the posttreatment state (on the right). In the pretreatment image, a voluminous lesion is observed in the endometrial cavity, with a high T2 signal, suggesting endometrial neoplasia. In the posttreatment scan, there is a significant reduction or disappearance of the lesion, indicating a positive therapeutic response. Additionally, there appears to be a reduction in endometrial thickening and an improvement in the uterine contour. These images are essential for monitoring treatment efficacy and guiding future strategies for fertility preservation and oncologic control. [file CROG-2026-5582003-s002.png]
